# Effects of Cryoprotectant Combinations on Post-Thawed Sperm Quality, Biomolecular Changes, DNA Methylation, and Pregnancy Rates in Boer Goat Semen

**DOI:** 10.3390/vetsci12020178

**Published:** 2025-02-17

**Authors:** Fareeda Suwor, Satoshi Kubota, Siriwan Nawong, Anone Thuangsanthia, Montequl Toyra, Pramote Paengkoum, Samorn Ponchunchoovong

**Affiliations:** 1 School of Animal Technology and Innovation, Institute of Agricultural Technology, Suranaree University of Technology, Nakhon Ratchasima 30000, Thailand; fareedasuwor@kkumail.com (F.S.); skubota@sut.ac.th (S.K.); pramote@sut.ac.th (P.P.); 2 Synchrotron Light Research Institute (Public Organization), Nakhon Ratchasima 30000, Thailand; siriwannawong@slri.or.th; 3 Embryo Transfer Technology Research and Development Center, Nakhon Ratchasima 30130, Thailand; biotech4@dld.go.th; 4 Faculty of Animal Husbandry, Bangladesh Agricultural University, Mymensingh 2202, Bangladesh; toyra2014@gmail.com

**Keywords:** sperm cryopreservation, egg yolk, soybean lecithin, synchrotron FTIR, DNA methylation, Boer goat

## Abstract

This study investigated the effects of various cryoprotectant combinations on post-thaw sperm quality, biomolecular changes, DNA methylation, and pregnancy rates using Boer goat semen. Synchrotron-based Fourier-transform infrared spectroscopy (SR-FTIR) was used to assess biomolecular changes. A Tris-based extender supplemented with 5% glycerol was used in combination with different concentrations of cryoprotectants, including 1% and 3% soybean lecithin and 10% and 18% egg yolk, with Andromed^®^ serving as the control. SR-FTIR analysis revealed that the combination of 5% glycerol and 18% egg yolk (T4) resulted in significantly higher levels of lipids, ester lipids, and secondary protein structures (α-helix) compared with those under the other treatments (*p* < 0.05). Analysis of the principal component analysis (PCA) score plot and correlation loadings revealed a positive association between the cryoprotectant combination of T4 and increased levels of lipids and ester lipids, as well as enhanced sperm motility, progressive motility, and viability. Furthermore, this combination achieved a pregnancy and parturition rate of 66.67%, which was notably higher than the rate achieved with Andromed^®^ (37.50%). Moreover, T4 did not show a significant difference in DNA methylation levels compared to Andromed^®^ and fresh sperm (*p* > 0.05). Overall, the results indicated that specific cryoprotectant combinations play a key role in enhancing the biomolecular and functional integrity of freeze-thawed Boer goat semen.

## Simple Summary

Boer goats, which are recognized for their rapid growth rate and adaptability to hot climates, are the most popular goat breed for meat production in Thailand. However, the use of artificial insemination with frozen semen in breeding faces significant challenges, primarily low conception rates. This is largely attributed to the quality of frozen semen because cryopreservation can damage sperm cells. This damage arises from changes in osmotic pressure and the formation of ice crystals both inside and outside the cells, which compromise sperm viability. To address these issues, this study explored the use of a Tris-based extender with an osmolality similar to that of Boer goat semen combined with a carefully selected cryoprotectant mixture. The formulation included a permeable cryoprotectant (5% glycerol) and nonpermeable cryoprotectants (1% and 3% soybean lecithin, and 10% and 18% egg yolk) to optimize the cryopreservation process. Synchrotron-based Fourier-transform infrared spectroscopy (SR-FTIR) was used to evaluate the effectiveness of the cryopreservation formulation. The results revealed a positive correlation between the combination of 5% glycerol and 18% egg yolk, leading to improvements in lipid and ester lipid levels as well as enhanced sperm motility, progressive motility, and viability. Overall, this study found that specific cryoprotectant combinations significantly improved both the biomolecular and functional integrity of freeze-thawed Boer goat semen.

## 1. Introduction

Thailand’s goat farming industry has experienced remarkable growth in recent years. The goat population increased by 2.4-fold from 650,000 in 2017 to 1,568,000 in 2023 [1]. Among the various breeds, Boer goats are distinguished by their rapid growth, high carcass yield, lean meat production, and adaptability to hot climates [2]. To enhance meat production, farmers frequently crossbreed Boer goats with other breeds to produce hybrids with desirable traits [3]. Despite this growth in the industry, the industry faces several challenges, including a reliance on imported goats, which increases production costs. Artificial insemination (AI) using frozen semen from high-quality breeds has become widely used to mitigate these challenges [4]. This technique has increased productivity, enhanced genetic quality, and helped control the spread of diseases [5]. However, the effectiveness of AI is hindered by low conception rates (38.70–43.80%), which are largely due to the limited availability of high-quality frozen semen [6,7]. Enhancing the quality and availability of frozen semen is crucial for sustainable goat farming in Thailand. However, the development of efficient cryopreservation protocols remains challenging. Freeze-thawing can cause irreversible damage to sperm DNA, proteins, and lipids, compromising their functional capacity and fertilization potential. Addressing these challenges is essential for improving semen quality and increasing reproductive efficiency in goat farming.

Cryoprotectants are crucial for mitigating cryodamage during sperm cryopreservation by preventing ice formation, reducing osmotic stress, and stabilizing cellular structures. Conventional cryoprotectants, such as glycerol and dimethyl sulfoxide (DMSO), are commonly used in this process. However, their use has certain limitations: higher concentrations may enhance protection but often lead to cytotoxicity [8,9], while lower concentrations fail to provide sufficient protection [10,11]. To address these limitations, recent studies have investigated the synergistic effects of combining permeating and nonpermeating cryoprotectants. For example, pairing glycerol with nonpermeating agents, such as soybean lecithin or egg yolk, has been shown to improve the osmotic balance and preserve the cellular structures during freezing [10,11,12]. These combinations have been shown to enhance sperm motility, viability, and cellular integrity in various goat breeds, including meat and milk-producing breeds; they key meat breeds included Mahabadi goats [12,13,14,15], Sarda goats [16], and Damascus goats [17], as well as native meat breeds such as Gaddi [18], Chongming White [10], Malabari [19], and Yunshang Black [20]. Significant improvements have been achieved using these approaches with milk breeds such as Saanen [11]. Although combined cryoprotectants have shown efficacy across a range of goat breeds, their effectiveness depends on species-specific factors and the optimization of concentrations to avoid cytotoxic effects. Despite these advances, the application of such strategies in Boer goats remains largely unexplored, representing a critical research gap.

Synchrotron-based Fourier-transform infrared spectroscopy (SR-FTIR) is a powerful technique for analyzing biomolecular changes in sperm, such as alterations in lipids, proteins (amides I and II), and nucleic acids [21,22]. This advanced method offers several advantages: it enables rapid analysis, requires only a small volume of semen, minimizes the use of chemical reagents, and provides detailed insights into molecular-level changes. Previous studies have demonstrated a significant correlation between molecular alterations and fertility in various species. For example, Pachetti et al. [22] reported that an increase in α-helix content correlated with sperm capacitation in human sperm, while a rise in β-sheet content was associated with reduced motility and viability. Similarly, Oldenhof et al. [21] investigated the chromatin and DNA structures in stallion sperm and found that reduced fertility was associated with an increase in abnormal sperm morphology. They observed changes in the IR bands of the DNA backbone and proteins, which resulted from oxidative damage and chromatin condensation. Building on these findings, this study aimed to utilize SR-FTIR to monitor the biomolecular changes in sperm to enhance the precision of selecting optimal cryoprotectant combinations. However, research on the biomolecular changes in goat semen using SR-FTIR remains limited, highlighting the need for further investigation.

Moreover, the freeze–thaw process significantly affects epigenetic mechanisms, particularly DNA methylation, which is crucial for regulating gene expression and sperm functionality. Cryopreservation alters DNA methylation patterns and potentially disrupts chromatin structure, capacitation, sperm function, and embryonic development [23,24]. For example, increased cytosine methylation has been observed in stallion sperm after cryopreservation [25]. These aberrant methylation patterns have been linked to impaired fertilization, abnormal embryo development, and a heightened risk of offspring developing epigenetic diseases [26,27]. In goats, Heidari et al. [28] observed that the freeze–thaw process adversely affected several aspects of sperm quality, including membrane functionality, normal morphology, mitochondrial activity, acrosome integrity, and DNA methylation in buck sperm. However, research on DNA methylation in goat semen is limited, highlighting the need for further investigations in this area. Therefore, this study aimed to investigate the effects of combining permeating and nonpermeating cryoprotectants on post-thawed sperm quality and biomolecular changes using SR-FTIR, DNA methylation, and pregnancy rates in Boer goats. These findings provide valuable insights into the interactions among cryoprotectants, sperm biomolecular stability, and epigenetic modifications, thereby significantly contributing to advancements in livestock cryobiology and reproductive biotechnology.

## 2. Materials and Methods

### 2.1. Animals, Semen Collection, and Primary Evaluation

Five healthy and mature semi-donor Boer goats with a body score of 3 and a mean weight of 60–70 kg goat^−1^ were held at the experimental goat farm of the Institute of Agricultural Technology, Suranaree University of Technology (SUT), Thailand. Throughout this study, the animals were maintained under the same management conditions and were fed twice a day with 18% protein per 2.5% body weight (based on dry matter) per goat per day. Fresh water was provided ad libitum. The Animal Ethics Committee of the Institute of Research and Development, Suranaree University of Technology reviewed and approved this study (approval No. U1-02632-2559).

Semen was collected twice a week from each buck using an artificial vagina (IMV Technologies, L’Aigle, France) and a female goat as a mount. Within 10 min of collection, the semen was transferred to a laboratory and kept in a water bath at 37 °C. The quality of individual goat semen samples was evaluated based on sperm concentration and motility. Sperm motility was assessed by computer-assisted semen analysis (CASA) with the HTT-CEROS system (Hamilton Thorne Biosciences, Beverly, MA, USA). Only semen samples with an initial motility percentage of >90% were accepted, pooled, and cryopreserved. The sperm concentration was determined using a photometer (SDM 1, Minitab International AG, Tiefenbach, Germany). Sperm concentrations ranging from 1100 to 6500 million sperm mL^−1^ were used for cryopreservation.

### 2.2. Chemicals

All chemicals used in this study were of reagent grade and purchased from Sigma-Aldrich (St. Louis, MO, USA) and Merck (Darmstadt, Germany).

### 2.3. Sperm Cryopreservation

Fresh sperm was collected from five Boer goats. Only sperm samples with a motility greater than 90% and concentrations ranging from 3416 to 4190 million sperm mL^−1^ were pooled and used for cryopreservation. A Tris-based extender containing 250 mM Tris, 88.5 mM citric acid, 69.38 mM fructose, and 300 µL of an antibiotic solution (Lincospecs, Viet Tri, Vietnam) was prepared. The pH and osmotic pressure of the extender were adjusted to match the physiological conditions of Boer goat semen, with a pH of 6.08 ± 0.01 and an osmolality of 321 ± 1.81 mOsm kg^−1^. The Tris-based extender was supplemented with 5% glycerol, and cryoprotectant combinations at different concentrations were investigated: 1% and 3% soybean lecithin as well as 10% and 18% egg yolk. To evaluate egg yolk as a cryoprotectant, the pooled semen was washed with lactated Ringer’s solution (General Hospital Products (Public) Co., Ltd., Pathum Thani, Thailand) and centrifuged at 1500× *g* for 3 min to remove seminal plasma. The supernatant was discarded, and sperm dilution was performed. Andromed^®^ (a commercial diluent, Mumbai, India) was used as the control.

**Figure 1:**
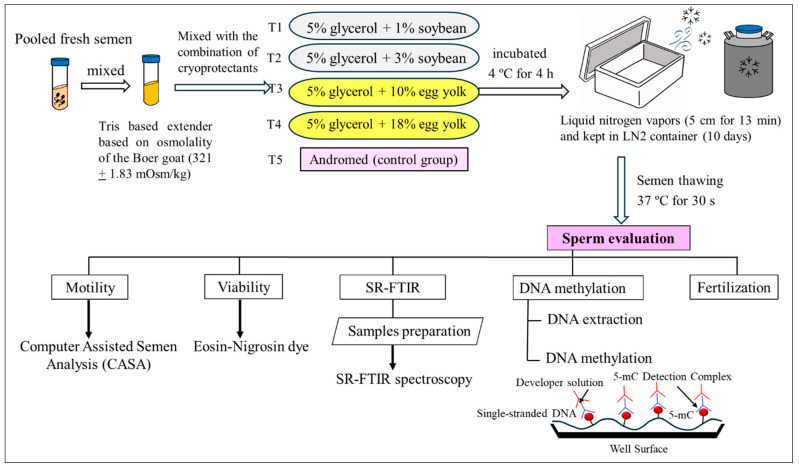
A schematic diagram of the experimental schedule.

Sperm samples were collected six times for each treatment, with a final concentration of 150 million sperm per straw. Then, the mixtures were placed inside a precooled cooling cabinet maintained at 4 °C for 4 h, following the method described by Abedin et al. [29]. The mixtures (230 µL) were loaded into 250 µL French straws using a filling nozzle with a 20-pin suction cap (0.25 mL; IMV Technologies, L’Aigle, France) and sealed with sealing powder (IMV Technologies, L’Aigle, France). For vapor freezing, the straws were placed in vapor (5 cm above liquid nitrogen) for 13 min. Afterward, the straws were plunged into a liquid nitrogen container at −196 °C. After storage for 15 days, the frozen sperm samples were thawed individually at 37 °C in a water bath for 30 s. Subsequently, the sperm motility, progressive motility, viability, and biomolecular properties of the samples were evaluated using SR-FTIR, and DNA methylation was measured. The treatment yielding the highest-quality frozen semen was selected for the fertilization of female Boer goats. A diagram of the experimental setup is shown in Figure 1.

### 2.4. Assessment of Sperm Motility, Progressive Motility, and Viability

Straws from each treatment group were thawed to assess sperm motility, progressive motility, and viability. Each treatment group consisted of six straws. A 10 µL aliquot of thawed sperm (containing approximately 6.52 million spermatozoa) was diluted with 180 μL of extender medium, and 3 μL of diluted semen was dropped onto a 2× CEL slide (Hamilton Thorne Biosciences, Beverly, MA, USA) and covered with a coverslip. Then, motility analysis was performed. Sperm motility was evaluated using CASA (HTT-CEROS; Hamilton Thorne Biosciences, Beverly, MA, USA). The CASA settings were optimized for the accurate detection and analysis of Boer sperm motility. The sperm-tracking parameters used for analysis are presented in Table 1.

Sperm viability was assessed using eosin–nigrosin staining. A 10 µL aliquot of thawed semen (containing approximately 6.52 million spermatozoa) was mixed with 50 µL of the stain on a glass slide. The mixture was evenly smeared across the slide and left to air dry for 3 min, and the viability was immediately checked. Viability was determined by examining 200 spermatozoa across five different microscopic fields using a bright-field microscope at 400× magnification. Unstained spermatozoa (live) appeared white, whereas spermatozoa stained pinkish (dead) were counted. The sperm viability was assessed utilizing the method described by Kwantong and Bart [30].

### 2.5. Assessment of Synchrotron-Based Fourier-Transform Infrared Spectroscopy (SR-FTIR)

A sperm sample with 150 million spermatozoa (230 µL) from each treatment group was transferred into a 1.5 mL Eppendorf tube, with three replicates per treatment. The samples were centrifuged at 8000 rpm for 3 min, and the seminal plasma and cryodiluent were removed. Then, the sperm sample was washed by adding 0.85% NaCl (0.5 mL), followed by centrifugation at 8000 rpm for 3 min. The washing step was repeated twice. After removing the NaCl solution, sterile distilled water (0.5 mL) was added, and the samples were centrifuged at 8000 rpm for 3 min. The rinsing step was repeated twice. Afterward, the distilled water was removed, and 50 µL of sterile distilled water was added to the sperm sample. Subsequently, 1 µL of the sperm suspension was placed onto a transparent BaF_2_ IR window and dried in a desiccator before analyzing the biomolecular properties across various treatments. Each treatment was performed in triplicate.

FTIR spectroscopy (Vertex 70) coupled with an infrared microscope (Hyperion 2000, Bruker Optics, Ettlingen, Germany) operated using OPUS software (version 7.5, Bruker Optics Ltd., Ettlingen, Germany) was used to analyze the spectral properties of the biomolecules. Spectral data were collected in the range of 3000–900 cm^−1^ with a spectral resolution of 6 cm^−1^ and 64 scans per measurement. For each sample, 100 spectra were collected per replicate to obtain 200 spectra. Spectral data were processed using Unscrambler X software (version 10.4; Camo Analytics, Oslo, Norway). The spectra were preprocessed by applying the Savitzky–Golay algorithm with a second derivative and 13-point smoothing to minimize the baseline variations. Vector normalization was applied to the spectral regions of 3000–2800 cm^−1^ and 1800–900 cm^−1^ to enhance the peak resolution and sharpen spectral features changes in these areas. The intensity of the focused biomolecular bands was analyzed and is expressed as a percentage of the integral area for the following regions: 3000–2800 cm^−1^ (CH_2_/CH_3_ lipids), 1750–1700 cm^−1^ (C=O ester lipids), 1700–1600 cm^−1^ (amide I), 1600–1500 cm^−1^ (amide II), and 1250–900 cm^−1^ (nucleic acids, carbohydrates, and fingerprint regions associated with DNA and RNA) using the guidelines provided by Pachetti et al. [22] and Pongsetkul et al. [31]. Secondary protein structures were quantified as percentages of α-helix, β-sheet, β-turn, and β-antiparallel conformations. This quantification was performed using curve-fitting techniques applied to the amide I region (1700–1600 cm^−1^) with the Gaussian and Lorentzian functions within OPUS 7.5 software (Bruker Optics Ltd., Ettlingen, Germany). Principal component analysis (PCA) was used to assess the variability within the spectral data.

### 2.6. DNA Methylation Analysis

The genomic DNA of the treated sperm was extracted using a Tissue Genomic DNA Extraction Kit (Favorgen Biotech Corp., Ping-Tung, Taiwan) following the manufacturer’s instructions with slight modifications. Briefly, 230 µL of frozen sperm containing 200 million sperm cells was prepared in triplicate for each treatment group. Samples were thawed at 4 °C and centrifuged at 6000 rpm for 5 min at the same temperature. The supernatants were discarded, and 1.5 mL of 0.9% NaCl was added to the sperm pellets. The mixtures were centrifuged at 14,000 rpm for 2 min at 4 °C. After the supernatant was removed, the sperm pellets were resuspended in 200 µL of FATG1 buffer, and 10 µL of RNase A (20 mg mL^−1^) was added.

The mixture was then pipetted and incubated at room temperature for 5 min. Subsequently, 20 µL of 10 mg mL^−1^ proteinase K was added to the sample and thoroughly mixed. The mixture was incubated at 56 °C for 30 min and vortexed every 10 min. After incubation, 10 µL of 1 M dithiothreitol (DTT) was added, and the sample was further incubated at 56 °C for an additional 30–60 min until it became clear. Samples were processed according to the manufacturer’s instructions. The quality and quantity of the genomic DNA were measured using a NanoDrop 2000c UV–Vis spectrophotometer (Thermo Scientific, Wilmington, DE, USA), and the integrity of the genomic DNA (gDNA) was assessed using 0.7% agarose gel electrophoresis.

The effects of the cryoprotectant combinations on global DNA methylation were analyzed using a global DNA methylation assay kit (5-methylcytosine [5-mC], colorimetry; Abcam, Cambridge, UK). DNA with a 260/280 ratio greater than 1.6 was used for global DNA methylation analysis. The assay was performed according to the manufacturer’s instructions using 100 nanograms (ng) of DNA per sample. The methylated DNA fraction was quantified by colorimetric analysis by measuring the absorbance at 450 nm using a microplate reader (Multiskan GO, Thermo Fisher Scientific, N.Y.R. Limited Partnership, Bangkok, Thailand). The percentage of methylated DNA (5-mC) in the spermatozoa was quantified using a standard curve regression equation and calculated using the following formula:

5-mC (%) = [(Sample OD − Negative Control OD)/Slope × S] × 100

where S represents the amount of input DNA (ng). Each treatment group comprised three replicates.

### 2.7. Estrus Synchronization and Artificial Insemination

Seventeen female Boer goats with normal reproductive cycles underwent estrus synchronization. Intravaginal CIDR^®^G goat devices, each containing 300 mg of progesterone (Zoetis South Africa Pty. Ltd., Sandton, South Africa; Zoetis Australia Pty. Ltd., New South Wales, Australia), were inserted and left in place for 13 days. Afterward, the goats received intramuscular injections of 200 IU of Folligon^®^ (pregnant mare serum gonadotrophin; PMSG, Intervet Ltd., Bangkok, Thailand) and 125 µg of Estrumate^®^ (prostaglandin F2∝; PGF2∝, Intervet Ltd., Bangkok, Thailand). A total of 48 h after the hormone injections, the CIDR^®^G devices were removed, and estrus was monitored every 4 h for 2 days. The seventeen female goats were divided into two groups to evaluate artificial insemination (AI). Group I was inseminated with semen frozen in a Tris-based extender containing 5% glycerol and 18% egg yolk, whereas Group II was inseminated with semen frozen in Andromed^®^. AI was performed twice, at 36 and 56 h after CIDR^®^G removal, using the cervical technique. The pregnancy rate was determined 30–40 days after insemination using an ultrasound scanner (PL-3018I Digital Ultrasound Scanner, Xuzhou Palmary Electronics Co., Ltd., Xuzhou, China).

### 2.8. Statistical Analysis

The data were tested for normality using the PROC UNIVARIATE procedure and Shapiro–Wilk test. Statistical analysis was conducted using ANOVA in a completely randomized design using the SAS software package (SAS Institute, version 9.1, 2002, Cary, NC, USA). Duncan’s test was used to compare the treatment means at a significance level of *p* < 0.05. Results are presented as the mean ± standard error. The correlation between frozen semen quality and DNA methylation was analyzed using Pearson’s correlation analysis. Pregnancy and parturition were analyzed using descriptive statistics and are expressed as percentages (%).

## 3. Results

### 3.1. Effects of Cryoprotectant Combinations on Motility, Progressive Motility, and Viability of Frozen–Thawed Sperm in Boer Goats

The highest motility rate (50.60 ± 0.31%), progressive motility rate (30.75 ± 0.77%), and viability rate (62.00 ± 0.77%) were achieved using the combination of 5% glycerol and 18% egg yolk. These values were significantly higher than those achieved with the other treatments (*p* < 0.05, Table 2) but lower than those observed in the control group (Andromed^®^). The lowest motility and viability rates were observed in the treatment with the cryoprotectant combination of 5% glycerol and 1% soybean lecithin (*p* < 0.05; Table 2).

### 3.2. SR-FTIR Characteristics: Effects of Cryoprotectant Combinations on Biomolecular Changes of Frozen–Thawed Sperm Cells

**Figure 2:**
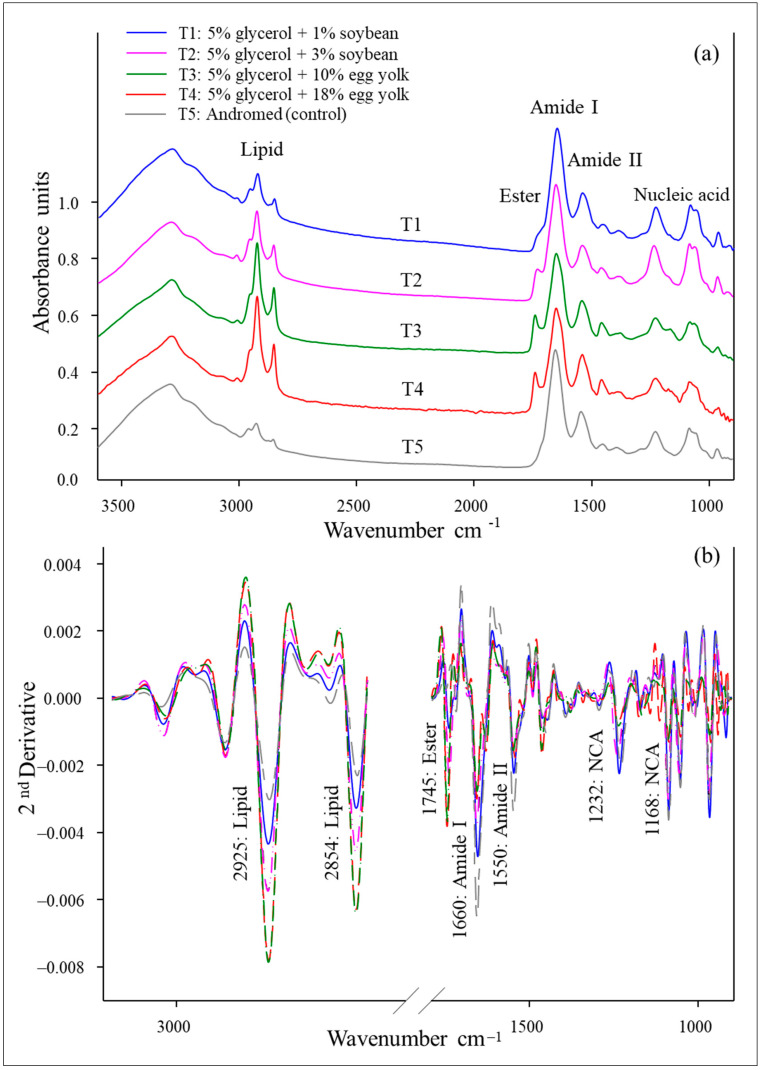
Average FTIR absorbance spectra in the spectral regions of 3000–900 cm^−1^ of the original spectra (**a**) and the second-derivative spectra (**b**) in Boer spermatozoa in the different experimental groups.

The average FTIR spectrum of frozen Boer goat spermatozoa from various treatments is shown, highlighting key biomolecular peak regions from 3000 to 900 cm^−1^ (Figure 2a). The spectra from each sample revealed distinct differences in peak heights and biomolecular ratios, including those of lipids (CH₂/CH₃ stretching lipids, 3000–2800 cm^−1^), ester lipids (1750–1700 cm^−1^), amide I proteins (protein secondary structures, 1700–1600 cm^−1^), amide II proteins (N-H bending and C-N stretching vibrations, 1600–1500 cm^−1^), and nucleic acids (carbohydrates, sugars, and DNA/RNA in the fingerprint region, 1250–900 cm^−1^) (Figure 2a). The second-derivative spectra are presented in Figure 2b, and their integrated areas (%) are summarized in Table 3. Among all the biomolecules, the amide I band (commonly observed in FTIR experiments) and lipids were the predominant components of the Boer goat sperm cells. Additionally, the use of a Tris-based extender supplemented with 5% glycerol and 18% egg yolk (T4) resulted in significantly higher percentages of lipids and ester lipids compared with those in the other treatments (*p* < 0.05), as shown in Table 3.

**Figure 3:**
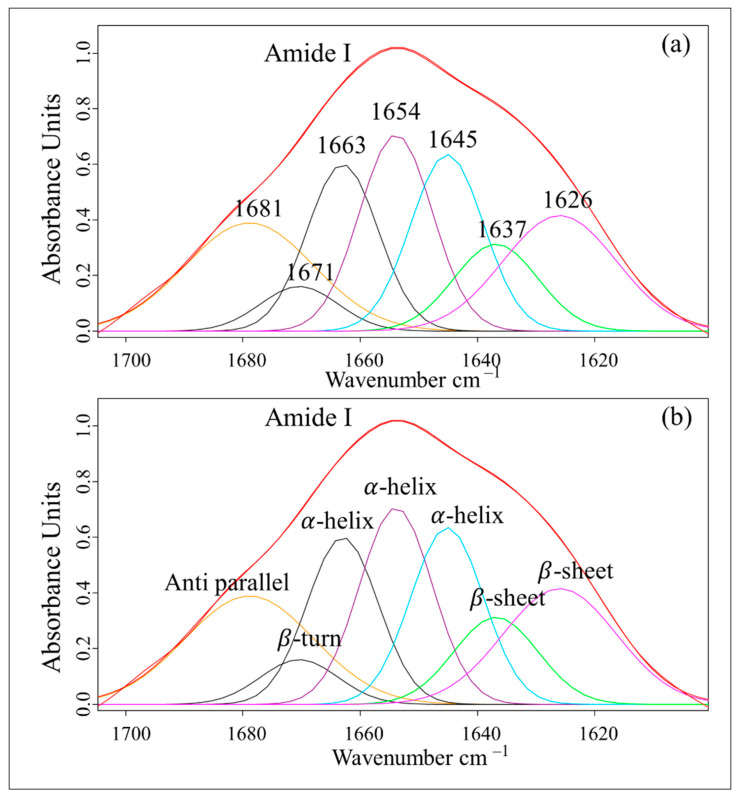
Curve fitting of the amide I band and secondary protein structure assignment in Boer spermatozoa from different experimental groups. In the spectral region of 1700–1600 cm^−1^, the wavenumbers 1640–1620 cm^−1^, 1670–1640 cm^−1^, 1678–1670 cm^−1^, and 1695–1680 cm^−1^ (**a**) correspond to the secondary protein structures of amide I, representing β-sheet, α-helix, β-turn, and β-antiparallel structures, respectively (**b**).

To quantify the changes in the most crucial spectra, the amide I spectra were subjected to curve-fitting analysis to determine the proportions of secondary protein structures, including β-sheet (1640–1620 cm^−1^), α-helix (1670–1640 cm^−1^), β-turn (1678–1670 cm^−1^), and β-antiparallel (1695–1680 cm^−1^) contents, as shown in Figure 3. The results indicated that T4 exhibited a significantly higher percentage of α-helices (49.74 ± 1.29) compared to the other treatments (*p* < 0.05), whilst the β-sheet content (30.21 ± 1.00) did not differ significantly from that in the other treatments (*p* > 0.05). Furthermore, the β-turn and β-antiparallel structures were not significantly different from those observed in the Andromed^®^ group (*p* > 0.05), as shown in Table 3.

**Figure 4:**
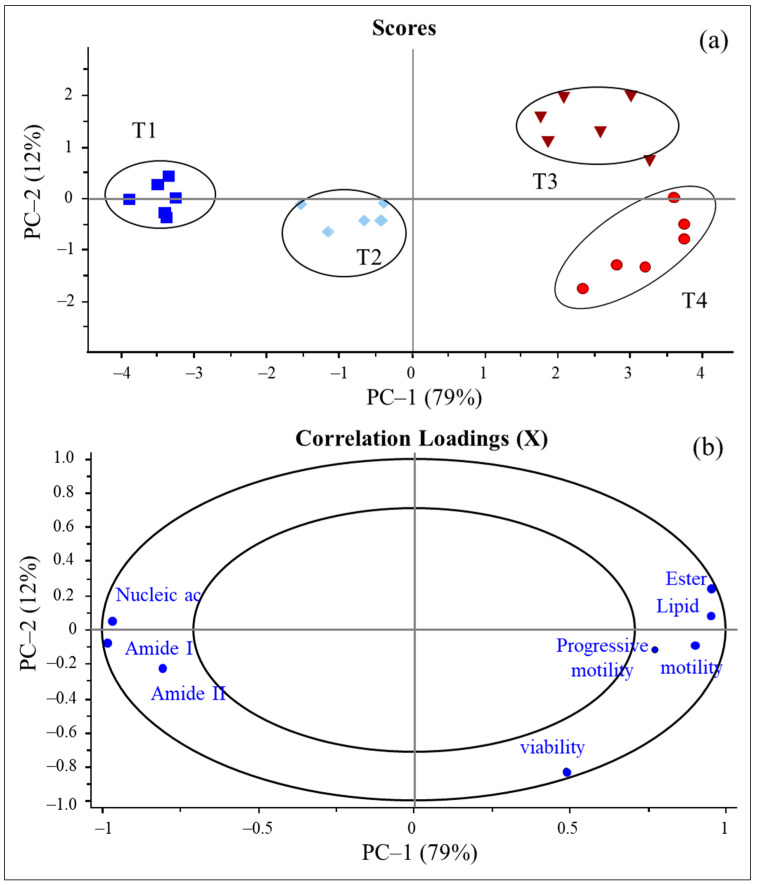
PCA scores plot (**a**) for PC1 versus PC2 showing the four different experimental groups, and the correlation loading plot (**b**) for PC1 versus PC2 illustrating total motility, progressive motility, and viability rate. Note: T1: 5% glycerol + 1% soybean lecithin; T2: 5% glycerol + 3% soybean lecithin; T3: 5% glycerol + 10% egg yolk; T4: 5% glycerol + 18% egg yolk.

The PCA score plot of the thawed sperm is illustrated in Figure 4a, from which we identified two distinct clusters among the treatment groups: one comprising the cryoprotectant combinations of glycerol and soybean lecithin (T1 and T2) and the other comprising the combinations of glycerol and egg yolk (T3 and T4). These clusters were separated along PC-1, which accounted for 79% of the variance (Figure 4a). The correlation loading (Figure 4b) shows the details of the biomolecules that differentiated thawed sperm among the treatment groups. The relationship between the PCA score plot (Figure 4a) and the correlation loadings (Figure 4b) revealed that the cryoprotectant combination of 5% glycerol and 18% egg yolk (T4) positively correlated with lipids, ester lipids, motility, progressive motility, and viability. In contrast, the cryoprotectant combination of 5% glycerol and 1% soybean lecithin (T1) exhibited a positive correlation with nucleic acids, amide I, and amide II but a negative correlation with total motility, progressive motility, and viability, as illustrated in Figure 4.

### 3.3. Effects of Cryoprotectant Combinations on DNA Methylation and Pregnancy Rates of Thawed Sperm

**Figure 5:**
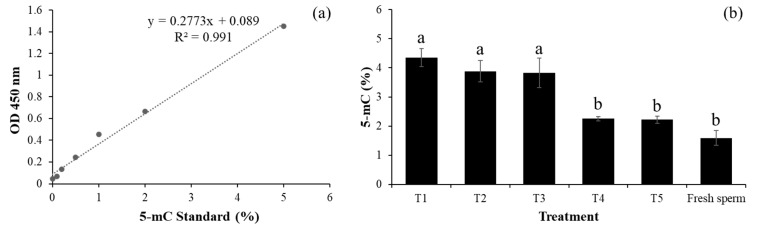
Standard curve for measuring 5-mC content (**a**) and the 5-mC amount, representing spermatozoa DNA methylation levels (**b**). Note: T1: 5% glycerol + 1% soybean lecithin; T2: 5% glycerol + 3% soybean lecithin; T3: 5% glycerol + 10% egg yolk; T4: 5% glycerol + 18% egg yolk; T5: Andromed^®^; T6: fresh sperm. Different letters indicate significant differences at *p* < 0.05.

The cryoprotectant combination of glycerol (5%) and egg yolk (18%) did not significantly alter the sperm DNA methylation compared with that of fresh sperm and that in the Andromed^®^ group (*p* > 0.05; see Figure 5b). However, significantly lower values were observed in the other treatments (*p* < 0.05). The correlations between thawed sperm quality and DNA methylation are shown in Table 4. Significant negative correlations were observed among sperm DNA methylation, motility (r = −0.897), and progressive motility (r = −0.918) (*p* < 0.05; Table 4).

Frozen semen supplemented with a combination of 5% glycerol and 18% egg yolk achieved a pregnancy and parturition rate of 66.67% (n = 6/9), whilst that in the Andromed^®^ group was 37.50% (n = 3/8), as shown in Table 5.

## 4. Discussion

### 4.1. Effects of the Cryoprotectant Combinations on the Motility, Progressive Motility, and Viability of Frozen-Thawed Sperm

Sperm cryopreservation and artificial insemination are essential reproductive technologies in the goat industry. The success of semen cryopreservation depends on multiple factors, including the composition of the freezing extender, selection of cryoprotectants, freezing and thawing protocols, and lipid composition of the sperm. In addition, the regulatory effects of seminal plasma play a crucial role in protecting sperm cells from damage during cryopreservation. The major lipid composition of goat spermatozoa primarily consists of phosphatidylcholine (PC; 36%), phosphatidylethanolamine (PE; 25%), and sphingomyelin (SM; 11%) [32]. In this study, an isosmotic Tris-based extender combined with 5% glycerol and 18% egg yolk (treatment 4, T4) enhanced the sperm motility, progressive motility, and viability of Boer goat semen compared with the other treatments. However, these parameters were significantly lower than those observed in the control group, in which Andromed^®^ was used (*p* < 0.05; Table 2). The iso-osmolality of the Tris-based extender (321 ± 1.83 mOsm kg^−1^) closely aligned with the osmolality of Boer goat seminal fluid (321 ± 4.42 mOsm kg^−1^). This congruence is crucial for maintaining sperm membrane integrity, as it prevents osmotic stress, which can compromise membrane permeability and fluidity during cryopreservation. Similarly, Paul et al. [33] reported that a hyperosmotic solution (450 mOsm kg^−1^) led to a noticeable loss of functional plasma membrane integrity compared with that under an iso-osmotic control (360 mOsm kg^−1^) in ram sperm. Sperm cell membranes are highly sensitive to cold shock and osmotic stress during cryopreservation. To mitigate cryodamage, a combination of cryoprotectants can stabilize the lipid and protein structures of the plasma membrane while preserving its integrity and fluidity [11,34,35]. Our findings indicated that 5% glycerol and 18% egg yolk enhanced sperm quality through complementary effects and that glycerol prevented intracellular ice formation, while egg yolk stabilized the membranes during freezing. Moreover, egg yolk, which is rich in phosphatidylcholine (73%), plays a crucial role in protecting sperm from cold shock during cryopreservation [36]. In contrast, soybean lecithin, which has a different phospholipid composition (30.8% PC, 32.5% PE, 27.9% phosphatidylinositol (PI), and 8.8% phosphatidic acid (PA)), cannot fully replace egg yolk in Tris-based extenders, as reported by Nguyen et al. [37,38]. In support of this, a study on ram semen revealed that egg-yolk-based extenders provided significantly better post-thaw sperm motility than soybean-lecithin-based extenders (*p* < 0.05) [39]. The high PC content in egg yolk enhances membrane fluidity and integrity, which are critical for sperm motility and capacitation, as we observed in Boer goat semen.

We observed that using a cryoprotectant combination of 5% glycerol and 10% egg yolk resulted in decreased sperm motility, progressive motility, and viability compared with those under the combination of 5% glycerol and 18% egg yolk. These findings are consistent with those of Rakha et al. [40], who reported that an extender containing 10% egg yolk combined with 0.6 mM polyvinylpyrrolidone (PVP) failed to adequately maintain rooster semen quality. In contrast, Kumar et al. [41] and Sharma et al. [18] successfully used a combination of 10% egg yolk and 6% glycerol to cryopreserve the semen of certain goat breeds, such as Jakhrana and Gaddi. Interestingly, while excessive egg yolk concentrations (e.g., 15% and 20%) are generally associated with physiological stress or imbalances that reduce sperm viability, Forouzanfar et al. [42] reported that a high egg yolk concentration (20%) combined with 7% glycerol was necessary to preserve motility and viability in ram sperm. These findings highlight the importance of optimizing extenders and cryoprotective agents to satisfy species-specific requirements. Variations in sperm tolerance to egg yolk concentrations and in cryoprotectant efficacy may explain these observed differences. Notably, the combination of 1% soybean lecithin and 5% glycerol resulted in the lowest motility and viability rates (*p* < 0.05; Table 2), likely owing to inadequate sperm membrane protection. Tailoring cryopreservation protocols for species-specific sperm characteristics is essential to improve post-thaw sperm quality. These findings align with those of Salmani et al. [12] and Sun et al. [10], who reported that low concentrations of soybean lecithin (0.5% and 1%) negatively affected the membrane integrity, viability, motility, acrosome integrity, and mitochondrial activity of freeze-thawed sperm obtained from Mahabadi and Chongming white goats.

### 4.2. Effects of Cryoprotectant Combinations on Biomolecular Changes and the Pregnancy Rates of Frozen–Thawed Sperm

SR-FTIR analysis demonstrated that the use of a Tris-based extender combined with 5% glycerol and 18% egg yolk (T4) resulted in significantly higher levels of lipids and ester lipids than the other treatments (*p* < 0.05) (Table 3). This enhancement was likely attributable to the high PC content (73%) in egg yolk, which is critical for maintaining sperm membrane integrity during cryopreservation. Egg yolk acts as a nonpermeable cryoprotectant, shielding sperm membranes from damage during freezing. Its protective effect is primarily derived from phospholipids, cholesterol, and low-density lipoproteins, which are key components for preserving membrane stability and functionality [43]. The composition of sperm plasma membranes, particularly the variety of lipid types, is critical for maintaining sperm function and integrity. Lipid metabolism is tightly regulated to support physiological cellular processes, including essential factors such as sperm motility, capacitation, acrosome reaction, and membrane fusion [44,45,46]. Alterations in lipid profiles have been linked to sperm dysfunction and potentially lead to infertility [22,47]. Therefore, optimizing the lipid content in cryopreservation protocols is crucial for maintaining sperm functionality and enhancing the success of cryopreservation. Our findings were consistent with the results of the PCA, which demonstrated a positive correlation between T4 (containing 18% egg yolk) and key biomolecules such as lipids and ester lipids, as well as sperm motility, progressive motility, and viability (Figure 4). These results suggest that specific cryoprotectant combinations, particularly those with higher concentrations of egg yolk, significantly influence the biomolecular composition and functional integrity of frozen–thawed Boer goat semen.

Monitoring the changes in the secondary protein structure of frozen–thawed semen is critical, particularly those in the α-helix content, as this common secondary structure provides both stability and flexibility, allowing proteins to dynamically interact with membranes, ions, and other molecules [22,46]. Our findings showed that T4 significantly increased the α-helix content to 49.74 ± 1.29%, whilst that in the control group was 45.36 ± 1.20% (*p* < 0.05, Table 3). This increase was associated with higher pregnancy and parturition rates in the control group (66.67% and 37.50%, respectively, Table 5). Our study found similar pregnancy and parturition rates. In contrast, Salama et al. [48] reported a lower parturition (goat kidding) rate than the pregnancy rate. They observed that the kidding rate improved in the 10% and 15% platelet-rich plasma (PRP) groups compared with that in the 5% PRP and control groups. Additionally, one goat in the 5% PRP group and two goats in the control group experienced abortion. This improvement may be attributed to the Tris-based extender mimicking the osmolality of seminal Boer goat fluid, thereby creating a biochemical environment conducive to maintaining sperm protein integrity. When combined with 5% glycerol and 18% egg yolk, this extender effectively protected the α-helix structures of the sperm proteins during cryopreservation. Glycerol, a penetrating cryoprotectant, reduces intracellular ice crystal formation, whereas egg yolk provides extracellular protection by stabilizing the cell membranes during freezing. These cryoprotectant combinations stabilized the α-helix structures in the proteins essential for sperm motility, acrosomal function, and oocyte binding. By preserving these proteins during the freeze–thaw process, sperm capacitation is enhanced, leading to higher fertilization rates. Our findings align with those of Pachetti et al. [22], who reported an increase in the α-helix content in capacitated sperm. Similarly, Shivanoor and David [46] observed a decrease in lipid content and α-helix structures in rat sperm exposed to high doses of cyanide (1.2 and 3.2 mg kg^−1^ BW). Notably, in our study, no significant effect of the cryoprotectant combinations on the percentage of β-sheets was observed (*p* > 0.05). This suggests that cryoprotectants, such as glycerol, soybean lecithin, and egg yolk, which are rich in phospholipids and antioxidants, provide robust protection for sperm membranes and proteins during cryopreservation. Sperm motility relies on ATP-driven flagellar activity, involving motor proteins, such as dynein and the enzymes involved in energy metabolic pathways [49]. These proteins predominantly consist of α-helices and intrinsically disordered regions rather than β-sheets, allowing their structural integrity to be better preserved during cryopreservation. Preserving these structures contributes to the maintenance of motility. Our findings contrast those of previous studies by Pachetti et al. [22] and Shivanoor and David [46], who reported that high percentages of β-sheet structures correlated with poor sperm quality in human and rat spermatozoa. These results underscore the critical role of α-helix stability in preserving sperm functionality and highlight the importance of carefully optimized cryoprotectant combinations to improve cryopreservation outcomes.

### 4.3. Effects of Cryoprotectant Combinations on the DNA Methylation Levels of Frozen–Thawed Sperm

During cryopreservation, oxidative and physical stress can induce epigenetic modifications, including increased 5-mC levels in specific genomic regions [24,25]. Our findings indicated that the cryoprotectant combination of 5% glycerol and 18% egg yolk did not significantly affect the global sperm DNA methylation levels compared with those of fresh sperm and Andromed^®^. In contrast, the other cryoprotectant combinations resulted in higher DNA methylation levels (Figure 5). Additionally, the decreases in sperm motility and progressive motility after cryopreservation correlated with increased DNA methylation levels, as shown in Table 4. This observation aligns with that of Zhou et al. [27], who investigated sperm cryopreservation in white pigs in Shanghai. They reported negative associations between spermatozoa DNA 5-mC and spermatozoa viability (R = −0.567), mitochondrial activity (R = −0.496), the integrity of spermatozoa plasma membranes (R = −0.789), and spermatozoa acrosomes (R = −0.769) (*p* < 0.05). Similarly, Aurich et al. [25] reported increased DNA methylation in horse spermatozoa after freezing, and Yeste [50] confirmed that cryopreservation enhances DNA methylation in boar semen.

These findings suggest that the increased DNA methylation levels resulting from cryopreservation may negatively affect sperm function by altering epigenetic stability, thereby affecting motility, viability, and structural integrity. The cryoprotectant combination of 5% glycerol and 18% egg yolk appeared to mitigate such epigenetic changes, potentially contributing to better post-thaw sperm quality than the other treatments. However, since we did not analyze the DNA methylation levels of individual genes, it remains unclear regarding whether there are highly methylated genes or whether a balance between hypermethylation and hypomethylation in different regions of the genome results in the lack of significant differences in global sperm DNA methylation levels.

Overall, these results demonstrate that specific cryoprotectant combinations significantly influence the biomolecular and functional integrity of freeze-thawed Boer goat semen. Among the tested combinations, the combination of 5% glycerol and 18% egg yolk was found to be the most effective. These findings provide insights that could contribute to the advancement of breeding programs for boar goats. However, to assess the impact of frozen semen quality on pregnancy and parturition rates more accurately, future studies should include a larger sample size of female goats.

## Figures and Tables

**Table 1:** Values of settings for assessing sperm motility using CASA (version 14).

Temperature (°C)	37
Apply sort	0
Frames acquired	30
Frames rate (Hz)	60
Minimum contrast	25
Minimum cell size (pixels)	4
Minimum static contrast	15
Straightness (STR) threshold (%)	80
VAP cutoff (µm/s)	5
Prog. min. VAP (µm/s)	20
VSL cutoff (µm/s)	20
Cell size (pixels)	4
Cell intensity	50
Static head size	0.72–8.82
Static head intensity	0.14–0.84
Static elongation	0–47
Slow cell motile	Yes
Magnification	1.92
Video frequency	60
Bright field	No
Chamber depth (µm)	20
Field selection mode	Auto

**Table 2:** Effects of cryoprotectant combinations on the motility, progressive motility, and viability of frozen–thawed sperm cells from Boer goats (mean ± SE).

Treatment	Motility (%)	Progressive Motility (%)	Viability (%)
T1	34.50 ± 0.95 ^e^	19.55 ± 0.88 ^d^	30.50 ± 0.99 ^e^
T2	39.20 ± 1.02 ^d^	20.10 ± 1.01 ^d^	47.00 ± 0.56 ^c^
T3	47.15 ± 0.44 ^c^	26.35 ± 1.11 ^c^	35.50 ± 0.92 ^d^
T4	50.60 ± 0.31 ^b^	30.75 ± 0.77 ^b^	62.00 ± 0.77 ^b^
T5	55.00 ± 0.61 ^a^	36.20 ± 0.97 ^a^	87.75 ± 0.82 ^a^

Note: T1, 5% glycerol + 1% soybean lecithin; T2, 5% glycerol + 3% soybean lecithin; T3, 5% glycerol + 10% egg yolk; T4, 5% glycerol + 18% egg yolk; T5, commercially available (Andromed^®^). Different letters in each column indicate significant differences at *p* < 0.05.

**Table 3:** Effects of cryoprotectant combinations on the biomolecule and protein structure contents of frozen–thawed Boer goat sperm (mean ± SE).

Biomolecules (Wavenumber cm^−1^)	Integral Area (%)				
	T1	T2	T3	T4	T5
Lipids (3000–2800)	23.20 ± 0.36 ^d^	26.56 ± 0.53 ^c^	39.00 ± 0.76 ^b^	41.28 ± 0.73 ^a^	10.32 ± 0.04 ^e^
Ester lipids (1750–1700)	1.54 ± 0.00 ^c^	1.58 ± 0.06 ^c^	4.71 ± 0.37 ^b^	5.52 ± 0.20 ^a^	0.00 ± 0.00 ^d^
Amide I (1700–1600)	40.91 ± 0.29 ^b^	40.71 ± 0.13 ^b^	30.83 ± 0.59 ^c^	30.54 ± 0.71 ^c^	57.83 ± 0.18 ^a^
Amide II (1600–1500)	9.55 ± 0.29 ^b^	7.81 ± 0.38 ^c^	8.80 ± 0.16 ^b^	9.49 ± 0.25 ^b^	13.23 ± 0.01 ^a^
Nucleic acid (1250–900)	24.81 ± 0.32 ^a^	23.34 ± 0.20 ^b^	16.67 ± 0.33 ^d^	13.17 ± 0.49 ^e^	18.62 ± 0.12 ^c^
**Secondary protein structure (% curve fitting)**					
β-sheet (1640–1620)	29.05 ± 0.54	30.72 ± 0.92	29.78 ± 1.00	30.21 ± 1.00	29.89 ± 0.97
α-helix (1670–1640)	42.73 ± 0.30 ^c^	43.85 ± 0.39 ^bc^	46.54 ± 0.90 ^b^	49.74 ± 1.29 ^a^	45.36 ± 1.20 ^bc^
β-turn (1678–1670)	6.71 ± 0.72 ^a^	6.09 ± 0.60 ^a^	6.84 ± 0.64 ^a^	3.72 ± 0.77 ^b^	5.68 ± 0.42 ^ab^
β-antiparallel (1695–1680)	21.51 ± 0.98 ^a^	19.34 ± 1.42 ^ab^	16.84 ± 0.39 ^b^	16.33 ± 0.44 ^b^	19.07 ± 0.86 ^ab^

Note: T1: 5% glycerol + 1% soybean lecithin; T2: 5% glycerol + 3% soybean lecithin; T3: 5% glycerol + 10% egg yolk; T4: 5% glycerol + 18% egg yolk; T5: Andromed^®^ (control). Different letters in each row indicate significant differences at *p* < 0.05.

**Table 4:** Correlation between sperm DNA methylation and the quality of frozen–thawed sperm from Boer goats.

Parameter	DNA Methylation	*p*-Value
Motility	r = −0.897 *	0.039
Progressive motility	r = −0.918 *	0.028
Viability	r = −0.875	0.052

Note: * indicates significant correlation at *p* < 0.05.

**Table 5:** The effects of the cryoprotectant combinations on the pregnancy and parturition rates of the thawed sperm.

Treatment	Inseminated Goats (n)	Pregnancy Rate % (n)	Parturition Rate % (n)
5% glycerol + 18% egg yolk	9	66.67 (6/9)	66.67 (6/9)
Andromed^®^	8	37.50 (3/8)	37.50 (3/8)

## Data Availability

The data presented in this study are available in this article.
